# Characterization of adverse reactions to benznidazole in patients with Chagas disease in the Federal District, Brazil

**DOI:** 10.1590/0037-8682-0150-2019

**Published:** 2020-01-27

**Authors:** Maria Katarine Costa Lucas Gontijo, Hilda Maria Benevides da Silva de Arruda, Elza Ferreira Noronha, Maria Inês de Toledo

**Affiliations:** 1 Universidade de Brasília, Faculdade de Ciências da Saúde, Brasília, DF, Brasil.; 2 Hospital Universitário de Brasília, Brasília, DF, Brasil.; 3 Universidade de Brasília, Faculdade de Medicina, Programa Pós-Graduação em Medicina Tropical, Brasília, DF, Brasil.

**Keywords:** Chagas disease, Benznidazole, Drug-related side effects, Adverse reactions

## Abstract

**INTRODUCTION:**

Benznidazole is used for treating Chagas disease (CD). This cross-sectional study aimed to characterize the adverse drug reactions (ADRs) of benznidazole at a public hospital in Brazil’s Federal District.

**METHODS:**

Medical records were analyzed and ADRs were categorized by type, intensity, seriousness, and causality.

**RESULTS:**

Of the 62 patients who started benznidazole treatment for CD, 41 (66%) presented with 105 ADRs; 23 (37%) discontinued the treatment. Most reactions were classified as probable (81%), severe (63%), serious (67%), and dose-dependent (56%).

**CONCLUSIONS:**

The high incidence of ADRs because of treatment withdrawal revealed the need for safer alternatives for CD treatment.

Worldwide, approximately seven million people live with Chagas disease (CD), 80% of whom have no access to diagnosis or treatment^1^ ^,^ ^2^. It is estimated that CD has resulted in 12,000 deaths annually^1^ ^,^ ^2^. 

Benznidazole, a nitroimidazole drug, has been adopted globally - including in the Brazilian Treatment Protocol and Therapeutic Guidelines for CD^1^ - as the first-line treatment, due to its more tolerable adverse drug reactions (ADRs), when compared to nifurtimox, the second-line option^1^ ^,^ ^3^.

Doses of 5 mg/kg/day for 60 days or 300 mg/day for up to 80 days are recommended for acute CD and some chronic presentations of the illness^1^. Dermatological, gastrointestinal, nervous system, musculoskeletal, hematological, and other manifestations may arise as ADRs during treatment^1^ ^,^ ^2^ ^,^ ^4^.

Since 2008, Brazil has been the patent holder of benznidazole, with the Laboratório Farmacêutico do Estado de Pernambuco as the sole producer of the drug until 2012^3^ ^,^ ^5^. With a half-life of roughly 12 h, the drug can be administered orally, with excellent gastrointestinal absorption. Maximum plasma concentration is attained within 2 to 4 h, with extra- and intracellular distribution, including the cerebrospinal fluid. As excretion is performed by the liver (~30%) and kidneys (60-70%), benznidazole is contraindicated for patients with severe disorders in these organs^1^ ^,^ ^3^ ^,^ ^6^. 

ADRs and long treatment periods have been associated with low adherence rates, impeding the evaluation of treatment responses^2^ ^,^ ^7^. Knowledge on the incidence and features of benznidazole-related ADRs, as well as, the impact of these reactions on users can help improve treatment outcomes and the management of ADRs.

The purpose of this investigation was to characterize suspected benznidazole-related ADRs in outpatients undergoing CD treatment at the Hospital Universitário de Brasília (HUB-UnB), a public teaching hospital in Brazil’s Federal District.

An observational, cross-sectional study of patients who started CD treatment with benznidazole at the HUB-UnB between December 2014 and March 2016 was conducted.

This study included patients with CD confirmed by indirect immunofluorescence and the hemagglutination assay (two tests based on different reagents) that initiated benznidazole treatment. The inclusion criterion was patient attendance to follow-up evaluations at 1, 6, and 12 months after initiation of treatment. Patients with incomplete information or those who missed follow-up appointments were excluded.

Suspected events were identified with the aid of a table of ADR triggers, based on published sources^1^ ^,^ ^2^ ^,^ ^3^ ^,^ ^4^ ^,^ ^5^ ^,^ ^6^ ^,^ ^7^ ^,^ ^8^ ^,^ ^9^ ^,^ ^10^ ^,^ ^11^ ^,^ ^12^. 

Information on sociodemographic variables (age, ethnicity, place of birth and of abode), clinical variables (disease stage and diagnosis date), pharmacoepidemiologic variables (dosage, ADRs, and comorbidities), and ADR management and evolution were collected from medical records.

ADR categorization was based on WHO concepts^13^ regarding reaction type, severity, and seriousness. The Naranjo algorithm^14^was applied to establish causal links between drug administration and adverse reactions.

Data were entered into Excel 2017 spreadsheets and exported to SPSS 22.0 software for descriptive statistical analysis of frequencies and for the construction of a cross-reaction table.

The project was approved by the Research Ethics Committee of the Universidade de Brasília School of Medicine (permit 1521680).

Six of the 68 patients failed to meet the inclusion criterion on the grounds of insufficient information about treatment. Thirty-four (54.8%) completed treatment with no dosage changes, 5 (8.1%) completed treatment with dosage adjustments, and 23 (37.1%) discontinued treatment. 

Median age of the 62 patients (46 females, 66.7%) included in this study was 45.4 years. Thirty-five (56.4%) self-identified as mixed black and white, 39 (62.9%) were originally from Northeast Brazil, and 35 (56.4%) lived in the Federal District. CD clinical presentation during treatment was indeterminate in 43 (69.3%) subjects. Mild cardiomyopathy was present in 10 (16.1%). Earliest and latest diagnoses were established in 1980 and 2015, respectively.

Thirty-five patients had at least one comorbidity - systemic arterial hypertension in 15 (24.1%); dyslipidemia in 10 (16.1%) - and 31 (50%) were in use of long-term control medications, even while taking benznidazole, of which anti-hypertensives (angiotensin II receptor blockers and thiazide diuretics) and antidepressants were the most reported (Table 1).

**TABLE 1:** Sociodemographic and clinical data of 62 patients with Chagas disease who started treatment with benznidazole at the Hospital Universitário de Brasília between December 2014 and March 2016.

Variable	Frequency			
	Total of patients		Patients with ADRs	
	*%*	n	*%*	n
**Number of Patients**	100	62	100	41
**Age (years)**				
30-45	59.7	37	73.2	30
>45	28.6	25	26.8	11
**Sex**				
Female	74.2	46	75.6	31
Male	25.8	16	24.4	10
**Reported skin color/ethnicity**				
Mixed black and white	56.4	35	58.5	24
White (Caucasian)	21	13	19.5	8
Black (African Brazilian)	12.9	8	14.6	6
Yellow (East Asian)	1.6	1	2.4	1
**Place of birth**				
Midwest Brazil	11.3	7	14.6	6
Northeast Brazil	62.9	39	70.7	29
Southeast Brazil	24.2	15	14.6	6
**Place of abode**				
Federal District	56.4	35	58.5	24
Elsewhere in Brazil	38.7	24	36.6	15
**Clinical presentation**				
Indeterminate	69.3	43	56.1	23
Cardiac, mild	16.1	10	31.7	13
Digestive	6.5	4	4.9	2
Mixed	3.2	2	2.4	1
**Long-term control medications**				
Yes	50	31	43.9	18
No	38.7	24	41.4	17
**Comorbidities**				
Yes	56.4	35	48.7	20
No	32.3	20	34.1	14

Thirty-seven (59.7%) patients took benznidazole at a concentration of 300 mg/day. Mean duration of benznidazole treatment was 47 days, even though the drug had been prescribed for a period of 60, 90, or 180 days.

Forty-one subjects (66.1%) reported suspected ADRs that were later associated with benznidazole use. Among these patients, the duration of treatment ranged from 1 to 90 days (mean, 36 days). 

A total of 105 suspected ADRs (range, 1-10 per patient; mean, 2.7) were identified, encompassing 41 types. Pruritus, nausea, unspecified allergic reaction, exanthema, and epigastralgia were the most frequent reactions. Dermatological reactions predominated, manifesting in 18 (81.8%) of the 22 (53.6%) patients with ADRs who discontinued treatment (Table 2). Other ADRs, including anorexia, anxiety, dyspnea, tachycardia, infection, presyncope, blurred vision, arthralgia, and edema, were also reported. 

**TABLE 2:** Frequencies of affected systems and principal adverse reactions to benznidazole in 62 patients with Chagas disease who started treatment at the Hospital Universitário de Brasília between December 2014 and March 2016.

System	Frequency		Principal	Frequency	
	%	n	reactions	%	n
Dermatological	48.4	30	Pruritus	25.8	16
			Unspecified allergic reaction	14.5	9
					
Gastrointestinal	22.6	14	Nausea	14.5	9
			Epigastralgia	9.6	6
					
Nervous	30.0	13	Vertigo	8.1	5
			Paresthesia	4.8	3
					
Musculoskeletal	14.5	9	Myalgia	4.8	3
			Asthenia	4.8	3
					
Other	8.1	5	Headache	4.8	3
			Fever	3.2	2

Thirteen (31.7%) of the 41 patients with suspected ADRs underwent symptomatic treatment, predominantly with anti-allergic drugs, mainly antihistamines (12 drugs, 7 patients), followed by corticoids (4 drugs, 4 patients).

Most (56.2%) of the 105 suspected ADRs were type A (dose-dependent). Eighty-five (80.9%) were categorized as probable, 71 (67.5%) as serious, and 66 (63.4%) as severe (Table 3).

**TABLE 3:** Classification of 105 suspected adverse reactions to benznidazole, by causality (as per the Naranjo algorithm), intensity, seriousness, and type, in 62 patients with Chagas disease who started treatment at the Hospital Universitário de Brasília between December 2014 and March 2016.

	Frequency		
Reaction profile	%	n
**Causality**		
Probable	80.9	85
Possible	12.4	13
Definite	3.8	4
Doubtful	2.9	3
**Intensity**		
Severe	63.4	67
Mild	19.5	20
Moderate	12.2	13
**Seriousness**		
Serious	67.6	71
**Type**		
A (dose-dependent)	56.2	59
B (dose-independent)	43.8	46

Mean age of patients with suspected ADRs who discontinued treatment was 44 years. Mean length of treatment was approximately 15 days. Patients who discontinued benznidazole had 23 suspected ADRs on average with rash (9), nausea (5), unspecified allergic reaction (5), and exanthema (3) as the principal causes. 

The present study revealed an association between most of the suspected ADRs and benznidazole use. Suspected ADRs affected 66% of patients. ADRs contributed significantly to the 37% treatment withdrawal rate. 

Chagas heart disease, described as the prevalent chronic symptomatic presentation of CD^2^, affected 16% of subjects in the present study. Most participants lived in Carinhanha (Bahia state), an area of vector-borne transmission risk^2^ ^,^ ^15^. Systemic arterial hypertension was the principal comorbidity. In Brazil, cardiopathies and circulatory diseases were the most frequent comorbidities in CD patients who died in 1999-2007^3^.

In a systematic review of benznidazole-related reactions, Viotti et al.^8^ found an incidence of ADRs of any intensity of roughly 50% among chronic patients (mean age, 36.9 years), with allergic dermatitis as the most common manifestation (25.3%) and treatment withdrawal rates of 12-18%. In a prospective observational study of Latin Americans who sought treatment at a hospital in Barcelona, Pinazo et al.^6^ reported that 98% of 57 patients (mean age, 37 years) evaluated for 60 days had ADRs, which accounted for treatment discontinuation in 20.5% of these individuals.

In a multicenter, triple-blind controlled investigation of 249 patients, Sosa-Estani et al.^10^ found that 57% of patients developed benznidazole-related ADRs, with morbilliform exanthema, pruritus, headache, and epigastralgia as the predominant symptoms. Treatment was discontinued in 17.7% of subjects. 

Similar results were found by Pinazo et al.^7^ among 105 patients, 57.1% of whom developed ADRs, with headache (56.2%) and pruritus (43.4%) as the principal manifestations. Of the subjects investigated, 16.2% did not complete their treatment. 

In a cohort study of 33 patients receiving benznidazole at 5 mg/kg/day for 30 days, Fabbro et al.^12^ found that maculopapular erythema and pruritus were the predominant ADRs, affecting 27% of the cohort. 

Evaluating 32 patients treated with benznidazole for 60 days, Pontes et al.^16^ found ADRs in 87.5%. Treatment was discontinued in 25%. Pruritus was the principal reaction (50%), followed by paresthesia (43.8%). ADRs contributed significantly to treatment withdrawal, and 53.7% of patients who developed adverse reactions stopped treatment. 

In the present investigation, ADRs (80.9%) had a probable causal link with benznidazole, while this link proved definite for only 2.9% of cases. Of the ADRs, 63.4% were categorized as severe and 67.6% as serious. No lethal reactions associated with benznidazole were reported. Pontes et al.^16^ reported 60.7% of ADRs as probable, 28.5% as possible, and 3.6% as definite. Of the total cases, 73% were mild and 27% moderate. No serious or lethal reactions were reported.

Owing to its cross-sectional retrospective design, this study has limitations concerning the records of suspected ADRs, even if data were reviewed using the clinical cohort database. This limitation can hinder comparisons with prospective studies, which can identify ADRs in higher numbers. In addition, ADR classification has not been uniform across previous investigations. 

The high incidence and relevance observed for benznidazole-related ADRs as a cause of treatment withdrawal reveals the need for safer alternatives to the available options of CD treatments. However, benznidazole remains the first-line option. Moreover, the results of this investigation highlight the need to improve ADR management through the adoption of protocols for adjunctive symptomatic treatment and early evaluation of patients initiating treatment.
